# On the Powers of Absorption of Mucous Membrane of Urinary Bladder

**Published:** 1891-12

**Authors:** 


ON THE POWERS OF ABSORPTION OF THE MUCOUS MEMBRANE OF THE URINARY BLADDER IN HEALTH. By Dr. B. London, Carlsbad, Austria.

This short essay gives the original researches of Dr. London upon the above subject, and show very laborious effort to determine the question. From his investigations, the Doctor comes to the conclusion "that the power of absorption of the mucous membrane of the bladder in comparison to the same membrane of other organs is very slow and insignificant; which circumstance, according to other authors as well, is to be attributed to the very considerable thickness of the bladder epithelium."

In his investigations as to the part played by the epithelium in this resistance to absorption he finds that "there is neither a displacement nor a break of continuity in the epithelial stratum whatever may be the degree of dilatation and consequent change of capacity of the bladder, but owing to the extraordinary elasticity with which the epithelial stratum is endowed, there is a decided change of shape in the individual cells, proportionate to the change of the entire epithelial structures."

Dr. London is well known as the medical adviser to many Americans who go to Carlsbad for the sake of the baths. W. M. J.

